# Inhaled RNA Therapeutics for Obstructive Airway Diseases: Recent Advances and Future Prospects

**DOI:** 10.3390/pharmaceutics13020177

**Published:** 2021-01-28

**Authors:** You Xu, Aneesh Thakur, Yibang Zhang, Camilla Foged

**Affiliations:** 1 Department of Pharmacy, Faculty of Health and Medical Sciences, University of Copenhagen, 2100 Copenhagen, Denmark; you.xu@sund.ku.dk (Y.X.); aneesh.thakur@sund.ku.dk (A.T.); zhangyb@ujs.edu.cn (Y.Z.); 2 Department of Pharmaceutics, School of Pharmacy, Jiangsu University, Zhenjiang 212013, China

**Keywords:** asthma, chronic obstructive pulmonary disease, drug delivery, dry powder, inhaled medicine, quality attributes, RNA therapeutics

## Abstract

Obstructive airway diseases, e.g., chronic obstructive pulmonary disease (COPD) and asthma, represent leading causes of morbidity and mortality worldwide. However, the efficacy of currently available inhaled therapeutics is not sufficient for arresting disease progression and decreasing mortality, hence providing an urgent need for development of novel therapeutics. Local delivery to the airways via inhalation is promising for novel drugs, because it allows for delivery directly to the target site of action and minimizes systemic drug exposure. In addition, novel drug modalities like RNA therapeutics provide entirely new opportunities for highly specific treatment of airway diseases. Here, we review state of the art of conventional inhaled drugs used for the treatment of COPD and asthma with focus on quality attributes of inhaled medicines, and we outline the therapeutic potential and safety of novel drugs. Subsequently, we present recent advances in manufacturing of thermostable solid dosage forms for pulmonary administration, important quality attributes of inhalable dry powder formulations, and obstacles for the translation of inhalable solid dosage forms to the clinic. Delivery challenges for inhaled RNA therapeutics and delivery technologies used to overcome them are also discussed. Finally, we present future prospects of novel inhaled RNA-based therapeutics for treatment of obstructive airways diseases, and highlight major knowledge gaps, which require further investigation to advance RNA-based medicine towards the bedside.

## 1. Introduction

Obstructive respiratory diseases are chronic diseases of the airways, and chronic obstructive pulmonary disease (COPD) and asthma in particular are among the leading causes of morbidity and mortality, hence they represent substantial health and economic burdens [1,2]. COPD is a complex and multifactorial respiratory disease, which is caused by environmental factors, for example tobacco smoking, air pollution, allergens, genetic factors, and/or occupational risks [3]. Major pathological features of COPD are (i) obstructive bronchiolitis, (ii) emphysema, (iii) pulmonary hypersecretion, and (iv) small airway obstruction. However, the relative contribution of each of these conditions to COPD varies between patients [4]. Asthma is another complex and heterogeneous chronic inflammatory disorder, primarily affecting the airways of the lungs, and it is the most common chronic disease among children. It is characterized by the upregulation of genes involved in multiple inflammatory cascades [5]. Besides, 15–45% of the population with obstructive airway diseases display symptoms of both asthma and COPD, which is referred to as asthma-COPD overlap syndrome (ACOS) [6]. Patients with ACOS experience persistent airflow limitations with several features of both asthma and COPD, and the prevalence of ACOS has been shown to increase with age [7].

Ranked the third-leading cause of death, approximately 3.17 million people died from COPD in 2016, which represents an estimated 5% of all deaths worldwide [8]. Although asthma has a relatively low fatality rate, compared to other chronic diseases, still more than 339 million people suffered from asthma globally in 2016, and asthma caused 0.4 million deaths [9]. Hence, the morbidity and mortality related to obstructive airway diseases are staggering and severely influencing the patients’ quality of life [10]. For example, the lining of the bronchial tubes swells during an asthma attack, which narrows the airways and reduces the air flow into and out of the lungs, hence leaving most patients unable to work. Patients with obstructive airway diseases are further bothered by frequent acute exacerbations, which are described as worsening of disease symptoms, requiring a change of daily medications, and, in severe cases, even hospitalization [11].

Asthma and COPD cannot be cured. However, adequate treatment can reduce symptoms, prevent disease progression, and increase the quality of life for patients suffering from the disease. Inhaled pharmacologic therapy represents a cornerstone in the treatment of COPD and asthma. Pulmonary administration is considered as one of the major routes for delivering drugs because it displays a number of advantages, compared to conventional administration of drugs via for example the oral route, e.g., (i) the therapeutic dose can be reduced, (ii) first-pass metabolism is circumvented, (iii) sustained drug delivery, and (iv) fewer systemic side effects [12]. Clinically, inhalation is widely accepted, and patients can use inhalation devices by themselves after appropriate training. This review discusses currently available inhaled medicines, novel inhaled medicines, inhalable RNA therapeutics and drug delivery systems, and manufacture of thermostable solid dosage forms. In addition, we discuss obstacles for the translation of inhalable solid dosage forms to the clinic.

## 2. Inhaled Medicines for Obstructive Airway Diseases

In the 1950s, the introduction of oral cortisone therapy for inflammatory disorders resulted in striking clinical efficacy. However, the subsequent launch of more potent oral corticosteroids, e.g., prednisone and prednisolone, was accompanied by unacceptable systemic adverse effects after long-term therapy, which led to the development of inhaled corticosteroids (ICSs), e.g., beclometasone dipropionate, budesonide, ciclesonide, flunisolide, fluticasone, mometasone [13]. The widespread use of ICSs has revolutionized the management of asthma, but most patients with COPD respond poorly to glucocorticoid treatment with little improvement in halting disease progression and reducing mortality, and high doses of ICSs may even increase the risk of pneumonia in patients with COPD [14]. Some patients with ACOS benefit from treatment with ICSs [6]. The recent development of long-acting β-agonists (LABA) and ICSs treatment has enabled the introduction of convenient once-daily inhaled products. However, improved therapies are required for treatment of patients with severe asthma, and more effective anti-inflammatory agents are needed.

Bacterial colonization of the lower airways has been reported for at least 50% of the patients with COPD (particularly patients with severe disease), and the same bacterial species associated with exacerbations are involved [15]. Macrolides display anti-inflammatory effects, independently of their antibiotic properties, and they are used to treat infections in the upper respiratory tract [16,17]. To avoid side effects, e.g., gastrointestinal symptoms and drug-induced QT prolongation (delayed ventricular repolarization), inhaled antibiotics like ciprofloxacin are currently being explored for clinical use (Clinical Trial No. NCT02661438). Liposomal formulations of inhaled ciprofloxacin are applied to enhance the residence time of antibiotics in the lungs [18].

Ipratropium bromide is a short-acting muscarinic receptor antagonist (SAMA), which was used for bronchodilator therapy of COPD during the 1970s. Subsequently, several long-acting muscarinic receptor antagonists (LAMA) have been introduced, e.g., aclidinium bromide, tiotropium bromide, glycopyrronium bromide, and umeclidinium bromide. Despite the availability of ICSs, antimicrobials, and LAMA agents, obstructive airway diseases are still recognized as complex and difficult-to-treat diseases. Single-inhaler triple therapy with once-daily fluticasone furoate/umeclidinium/vilanterol fixed-dose combination for the treatment of severe-to-very severe COPD has completed phase 4 clinical testing (maintenance treatment, Clinical Trial No. NCT03474081). Although offering greater convenience, these combination products do not appear to provide significant additional therapeutic benefits, and there is an unmet medical need for new disease-modifying drugs for asthma and COPD. Standard medicines for the treatment of COPD and asthma management, and their adverse effects, are summarized in Table 1.

## 3. The Fate of Aerosol Drugs after Deposition in the Lungs

**Figure 1:**
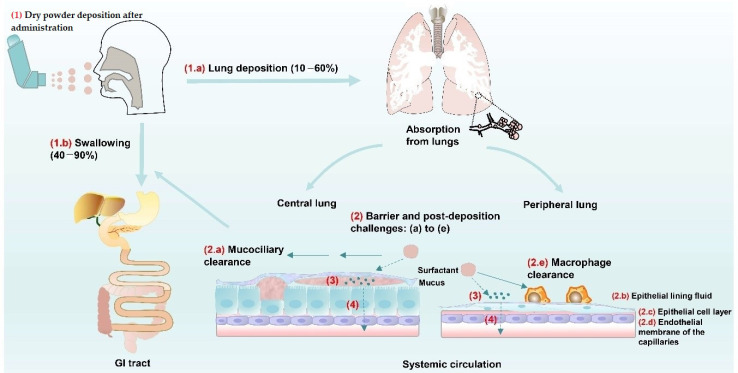
The fate of aerosol drugs after dosing. (**1**) Dry powder deposition after administration. Approximately 10–60% of the powder deposits in the lungs (**1.a**), while 40–90% is swallowed into the GI tract (**1.b**). (**2**) Barrier and post-deposition challenges after deposition in the lungs include (**2.a**) mucociliary clearance, (**2.b**) the epithelial lining fluid, (**2.c**) the epithelial cell layer, (**2.d**) the endothelial membrane of the capillaries, and (**2.e**) phagocytosis by macrophages. (**3**) Dry powder dissolves in the lung-lining fluid, and the API is subsequently released. (**4**) Absorption of the API across the pulmonary epithelium into the systemic circulation. Undissolved particles are cleared by (**2.a**) mucociliary clearance and (**2.e**) phagocytosis by macrophages. Abbreviations: API: active pharmaceutical ingredient; GI, gastrointestinal.

Developing inhaled medicines is inherently complex, requiring fine-tuning of drug formulation properties, including lung deposition, retention, dissolution, metabolism, and lung tolerability (Figure 1). The pulmonary route of administration can be used, as long as the disease by itself does not severely affect lung physiology, and/or causes breathing disturbances. The site at which particles deposit in the respiratory system is largely determined by the particle size. During the respiratory phase, particles must overcome the barriers related to lung geometry and the physiological conditions in the lungs, which are characterized by high relative humidity (approximately 90%) that may influence the particle size and hence interfere with the particle deposition pattern. Drugs must display a high potency to be suitable for inhalation therapy, because relatively small doses can be delivered (subnanomolar range). The transport of microparticles mainly depends on sedimentation and impaction mechanisms, and gravitational sedimentation governs particle deposition in the small conducting airways and in the gas-exchange regions of the lungs [26]. In contrast, the transport of particles, which are smaller than 0.5 µm in diameter, relies mainly on diffusion [27]. Generally, aerosols with a mass median aerodynamic diameter (MMAD) of 5–10 µm deposit in the large conducting airways and the oropharyngeal region [27]. Particles of 1–5 µm in diameter deposit in the small airways and the alveoli, and more than 50% of the 3 µm diameter particles deposits in the alveolar region. Particles smaller than 3 µm display approximately 80% chance of reaching the lower airways, and 50–60% deposit in the alveoli [28]. Hence, the optimal particle size for pulmonary delivery is in the range of 3 µm [29]. Airway deposition is also influenced by other factors, e.g., the inhalation waveform and the disease condition of the patient [30,31].

After deposition, particles undergo a number of processes, e.g., dissolution in the lung-lining fluid, lateral spreading at the air–water interface, and penetration within the fluid, which facilitates access across the various regions of the respiratory tract [32]. Barrier and post-deposition challenges in the lungs include (a) mucociliary clearance, (b) the epithelial lining fluid, (c) the epithelial cell layer, (d) the endothelial membrane of the capillaries, and (e) phagocytosis by macrophages [33] [Figure 1 (2)]. The cellular barriers and clearance processes influence the retention time of inhaled particles, which eventually determines the overall pulmonary drug bioavailability. In the upper respiratory tract, mucociliary clearance represents the dominant host defense mechanism [34]. Pulmonary mucus is a thick viscoelastic hydrogel layer, which is mainly composed of water and mucins [35]. Particles, which are deposited on the mucus layer, are inevitably pushed downward by surface forces [36], whereas mucociliary clearance transports particles deposited in the airways towards the mouth, where they are swallowed into the gastrointestinal system [37]. If the particles are rapidly cleared, they are less likely to be dissolved, followed by drug absorption in the airways [38].

The pulmonary surfactant layer constitutes a local barrier against infection. Mucus and inhaled particles are transported by the lung surfactant, which is synthesized by type II pneumocytes and secreted as multilamellar structures [39]. Pulmonary surfactant is composed of approximately 90% lipids and 10% proteins [40]. Several studies of particle-surfactant interactions have assessed the displacement of particles into the surfactant layer [36] and the effects that nanoparticles exert on the biophysical functionality of the surfactant film. These studies show that pulmonary surfactant promotes the displacement of particles, and particles affect the domain structure in lipid layers containing surfactant protein C [41]. If particles are not dissolved, they are likely to be recognized by alveolar macrophages. In the alveoli, particles interact with a thin lining layer (0.01–0.2 µm) [42] containing various mucins, lipids, and proteins. However, this lining layer does not represent a significant diffusion barrier to particle transport. The clearance by alveolar macrophages is closely associated with mucociliary clearance. Alveolar macrophages internalize particles with maximum efficiency when they display a diameter of 0.5–5 µm, which is in contrast to mucociliary clearance [43]. Nanoparticles with a diameter below approximately 260 nm have been shown to escape macrophage clearance [44]. Both clearance mechanisms are highly dependent on the properties of the mucus blanket in the upper and the central parts of the lungs, and the pulmonary surfactant layer constitutes the predominant defense mechanism in the peripheral lungs. The primary barrier for subsequent drug transport to the pulmonary capillary bed is the tight junctions, which are located between the alveolar epithelial cells.

After deposition, drug aerosols must dissolve in the lung-lining fluid for subsequent cellular uptake and/or absorption [45]. The thickness of the alveolar epithelium is approximately 0.1–0.5 µm, and it is highly vascularized and nonciliated, allowing for rapid drug absorption and onset of action [46]. Undissolved particles undergo clearance, resulting in fast removal from the body. Rapid clearance from the plasma after absorption can decrease the chance of drug exposure of nontarget tissues and organs and hence reduce systemic adverse effects. Therefore, the rate and extent of the dissolution process are the first challenges to improve absorption. The processes depend on the drug solubility, permeability, and solid-state properties, and the pharmacokinetic profile of a drug is strongly influenced by its formulation or carrier [47,48,49]. The amount of liquid available for dissolution represents another important factor. The total volume of the lung-lining fluid in human lungs has been estimated to 10–30 mL, and it is difficult to estimate the proportion of this total fluid volume that an aerosol particle is exposed to after deposition [28]. The combination of high drug potency and local pulmonary drug retention enables the use of low doses for inhalation therapy. Furthermore, components of the pulmonary surfactant layer can also affect the dissolution process and facilitate cell uptake, and many lung surfactant constituents are used as absorption enhancers [50].

## 4. Novel Inhaled Medicines for Obstructive Airway Diseases

**Figure 2:**
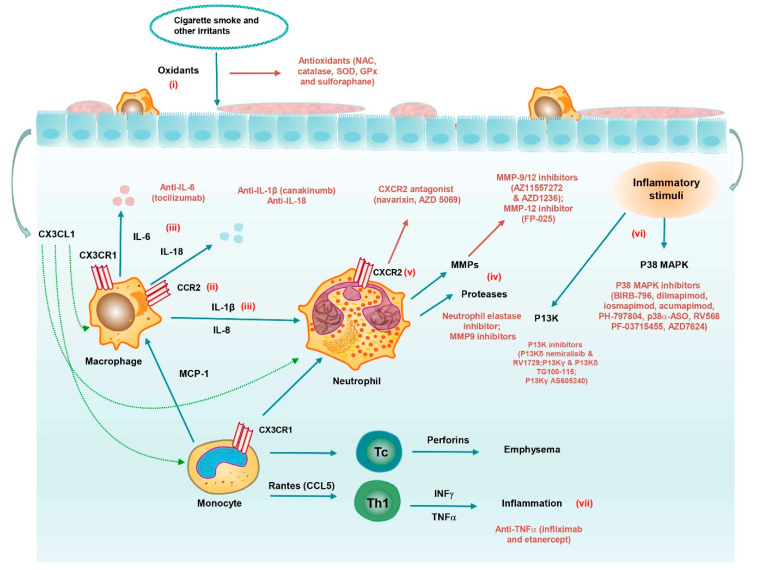
Novel therapeutic targets for COPD treatment. (**i**) Oxidative irritants, e.g., tobacco smoke, display a direct effect on epithelial cells, macrophages, and edematous basal membranes. (**ii**) Irritants activate macrophages to release several chemotactic factors, including CCL2, which attract inflammatory cells and act on CCR2 to attract monocytes. (**iii**) Macrophages also release proinflammatory cytokines, e.g., IL-1β and IL-18, following inflammasome activation and IL-6 secretion. (**iv**) Activated macrophages provide signaling to neutrophils, which cause an imbalance in the protease/antiprotease system and overexpression of MMPs. (**v**) Proinflammatory mediators attract neutrophils by binding to CXCR2. (**vi**) Inflammation stimulates the activation of PI3K, which leads to a reduction in sirtuin-1 levels; and stimulates the action of P38 MAPK, which increases the expression of the mucin genes, eventually leading to hyperplasia of goblet cells and submucosal glands. (**vii**) IFN-γ and TNF-α released by Th1 cells stimulate inflammation. Abbreviations: CCL: chemokines; CC-chemokine ligand; CXCR2: CXC- chemokine receptor 2; MAPK: p38 mitogen-activated protein kinase; MCP: monocyte chemoattractant protein; PI3K: phosphoinositide 3-kinase; Tc: cytotoxic T cell; Th1: T helper type 1 cell; TNF-α: tumor necrosis factor α.

**Figure 3:**
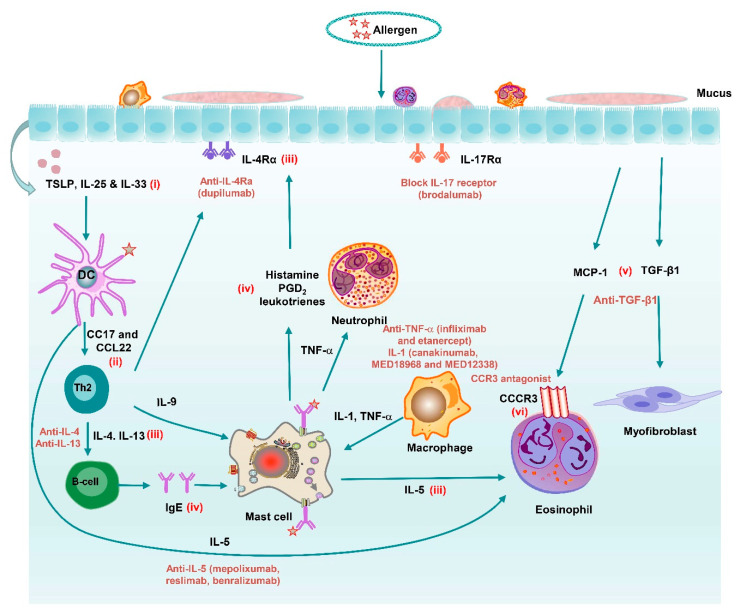
Novel therapeutic targets for asthma treatment. (**i**) Epithelial cells produce TSLP, IL-25, and IL-33, which are upstream cytokines in the eosinophilic response. (**ii**) Activated DCs release CCL17 and CCL22, which recruit Th2 cells. (**iii**) Th2 cells can secrete various cytokines, including IL-4, IL-5, IL-9, and IL-13. IL-4 promotes IgE synthesis by B cells. IL-4 receptor subunit α (IL-4Rα) causes the release of nitric oxide from airway epithelial cells. IL-5 acts on IL-5Rα to promote maturation and activation of eosinophils, and IL-9 is important for the integrity of mast cells. (**iv**) When triggered by allergen-IgE cross-linking, mast cells release histamine, PGD_2_, and leukotrienes, which lead to smooth muscle contraction and airway edema. (**v**) Fibrotic cytokines such as TGF-β can promote the proliferation of fibroblasts, the production and deposition of ECM proteins, and thereby promote subepithelial fibrosis and airway remodeling. (**vi**) Epithelial cells release eosinophils-attracting chemokines, e.g., CCL26, which attract eosinophils by binding to CCR3. Abbreviations: CCL: chemokines CC-chemokine ligand; CCR3: CC chemokine receptor 3; DCs: dendritic cells; ECM: extracellular matrix; Ig: immunoglobulin; IL: interleukin; IL-17Rα: IL-17 receptor α; IL-4 receptor subunit α (IL-4Rα); MCP: monocyte chemoattractant protein; PGD_2_: prostaglandin D2; TGF-β: transforming growth factor β; Th2: T helper type 2 cell; TNF-α: tumor necrosis factor α; TSLP: thymic stromal lymphopoietin.

The standard approaches for treatment of COPD and asthma do not offer additional therapeutic benefits and have not changed significantly since their introduction, e.g., no drugs can halt COPD disease progression (ICSs prevent disease exacerbations by approximately 20% reduction), and asthma remains uncontrolled in approximately 15% of the patients, who are refractory to corticosteroids. The unmet medical need continues to grow, and novel disease-modifying treatments for managing COPD and asthma include (i) antioxidants, (ii) mediator antagonists, (iii) kinase inhibitors, and (iv) RNA-based therapeutics (Figure 2 and Figure 3, and Table 2). The elastase emphysema and cigarette-smoke (CS) exposure animal model have largely been used for preclinical studies of these drugs. Clinical trials investigating the impact of new treatment strategies in patients with COPD or asthma are presented in Table 2.

### 4.1. Antioxidants

Oxidative stress is an important driver for the pathophysiology of obstructive airway diseases (Figure 2), which suggests that antioxidants may be effective therapeutics [95]. Therefore, some strategies target systemic and local oxidative stress using antioxidants/redox-modulating agents, or boost the endogenous levels of antioxidants. Commonly used nonenzymatic antioxidants include (i) glutathione [96], (ii) ascorbic acid [97], (iii) uric acid [98], and (iv) α-tocopherol [99]. Despite evidence for the beneficial role of N-acetylcysteine (NAC), the therapeutic efficacy of NAC/glutamines in the clinical management of COPD has remained controversial due to (i) its reduced bioavailability in an oral form, (ii) its acidic nature prohibiting its use in an inhaled form, and (iii) its low concentration in the lungs [100,101]. Enzymatic antioxidants mainly include (i) catalase, (ii) superoxide dismutase isomers, (iii) glutathione peroxidase, and (iv) glutathione-associated enzymes [102,103,104]. They exert strong anti-inflammatory effects on smoking-induced lung inflammation in animal models [55] and are currently undergoing clinical testing. Another therapeutic approach is to stimulate endogenous antioxidant defense mechanisms. Nuclear factor erythroid-2-related factor 2 (Nrf2) is a transcription factor activated by oxidative stress, which downregulates inflammation-associated production of reactive oxygen and nitrogen species [105]. Activation of Nrf2 has been shown to (i) protect mice from developing emphysema after chronic smoke exposure, (ii) decrease oxidative stress, (iii) increase proteasomal antiapoptotic cytoprotective responses, and (iv) improve bacterial phagocytosis and killing [106]. In human lung cells of COPD, Nrf2 activation has been shown to reduce oxidative stress and enhance bacterial clearance in macrophages [107]. However, sulforaphane, which was reported to activate Nrf2 and turn on several antioxidant pathways when administered for 4 weeks to patients with COPD, did not induce Nrf2 gene expression [56]. Although oxidative stress is an excellent target, antioxidant drug development relies heavily on the understanding of the complex oxidative stress mechanisms.

### 4.2. Mediator Antagonists

Many inflammatory mediators, including lipid mediators, cytokines, chemokines, and proteases, are involved in the complex inflammation of COPD and asthma. These mediators influence the recruitment and activation of inflammatory cells and the structural changes occurring during disease.

#### 4.2.1. Cytokine/Chemokine Inhibitors

The levels of many inflammatory cytokines, e.g., tumor necrosis factor-α (TNF-α), interleukin (IL)-1β, IL-4, IL-5, IL-6, IL-13, and IL-17, are significantly increased in obstructive airway diseases (Figure 2 and Figure 3) [108], and many of these cytokines can be inhibited using antibodies directed against the cytokines themselves, or their cognate receptors. Anti-TNF-α and anti-IL-1β and -6 antibodies have been shown to be effective in obstructive airway diseases [108]. The same clinical dose of TNF-α antibody used for the treatment of rheumatoid arthritis has a therapeutic effect on bronchial asthma, but this effect has not been confirmed in COPD patients. IL-5 can be targeted using blocking antibodies, e.g., mepolizumab and reslizumab, or its receptor (anti-IL 5 receptor α monoclonal antibody; benralizumab). Mepolizumab and reslizumab effectively reduce exacerbations in patients with severe asthma with increased sputum eosinophil counts [64,65], and might thereby contribute to controlling symptoms in asthma and reduce airway inflammation, and could potentially help to prevent remodeling. IL-13 is linked to structural remodeling in the airways, and hence might be relevant for the development of fixed obstruction in asthmatic patients [109]. IL-13 induces (i) mucus hypersecretion, (ii) airway fibrosis, and (iii) corticosteroid resistance, and anti-IL-13 therapies have shown some benefit in reducing exacerbations in asthma [71]. The antibody dupilumab blocks the common receptor for IL-4 and IL-13, i.e., IL-4Ra. Dupilumab has been shown to (i) reduce severe exacerbation rates, (ii) improve forced expiratory volume in 1 s (FEV1) and asthma control, and (iii) suppress type 2 inflammatory biomarkers in patients with uncontrolled, moderate-to-severe asthma with or without evidence of allergic asthma. The efficacy of dupilumab seems to be independent of eosinophil counts in patients with moderate to severe asthma [68,70]. Th2 cytokines are regulated by the transcription factor GATA3, which has been targeted by an inhaled oligonucleotide (SB010). SB010 is a DNA enzyme (DNAzyme), which is able to cleave and inactivate GATA3 mRNA. Inhaled SB010 significantly attenuated both the early-phase and late-phase asthmatic responses after allergen provocation, although it is challenging to inhibit this transcription factor intracellularly [72]. IL-17 signaling has been implicated in the development and persistence of asthma. However, blocking the IL-17 receptor (IL-17Ra) with brodalumab proved to be ineffective in patients with severe asthma [73].

Chemokines also play important roles in chronic inflammation and bronchial remodeling. Chemokines mediate various cellular processes by interacting with cell surface G-protein coupled receptors (GPCRs) [110]. GPCRs are generally classified into four groups, i.e., CCR, CXCR, XCR, and CX3CR. Chemokines influence the development and progression of obstructive airway diseases [111]. CCR1 has affinity for multiple chemokines, and CCR1 antagonists have been tested against autoimmune diseases in clinical trials. The inhaled CCR1 agonist AZD4818 was shown to be well tolerated in patients with COPD. However, there was no indication of a beneficial treatment effect, despite exposure as expected [74]. CCR3 is involved in eosinophil chemotaxis to chemokines, e.g., CCL11 (eotaxin), but CCR3 antagonists proved difficult to develop clinically [112]. Most attention has been focused on CXCR2 antagonists involved in neutrophil recruitment in response to CXCL8 and related CXC chemokines. Treatment of patients with severe asthma with oral navarixin reduced sputum neutrophil counts by approximately 40%, with some reduction in mild exacerbations. However, navarixin showed no improvement in FEV1, symptoms, or asthma control, and no reduction in sputum neutrophil activation markers [113]. The CXCR2 antagonist MK-7123 was shown to reduce COPD exacerbations and several sputum inflammatory biomarkers in smokers [75]. These studies suggest that CXCR2 antagonists reduce sputum neutrophilia, but with little or no clinical benefit. This might be because of the fact that the reduction in sputum neutrophil counts is insufficient, or that sputum neutrophil counts are not closely related to disease mechanisms. In addition, CXCR2 inhibition triggers side effects, e.g., promoting bacterial/ fungal infection and delaying wound healing [114].

#### 4.2.2. Inflammasome Inhibitors

The inflammasome, which is a multimeric protein complex, may be targeted therapeutically in inflammatory diseases. The inflammasome and its products are part of the innate immune system, which can be triggered to assist in the defense against invading pathogens. The inflammasome is a protein complex involved in caspase-1-dependent release of the proinflammatory cytokines IL-1 and IL-18 [115]. Emerging scientific evidence suggests persistent Nod-like Receptor 3 (NLRP3) inflammasome activation in patients with severe COPD and asthma [116,117]. Several randomized clinical trials, which target inflammasome-related effectors, have been performed in patients with moderate to severe COPD, but no significant therapeutic effect was observed (Table 2). Although the inflammasome is likely involved in the pathogenesis of COPD and asthma, further investigations are needed to clarify its role.

#### 4.2.3. Protease Inhibitors

Several proteases, which are not normally expressed in healthy tissue, display increased expression levels in COPD, e.g., matrix metalloproteinases (MMPs) and neutrophil elastase from alveolar macrophages, neutrophils, and bronchial epithelial cells [118]. This protease/antiprotease imbalance has been reported to be a key contributor to emphysematous changes [119]. MMPs, e.g., MMP-9 and MMP-12, constitute a large family of zinc-dependent proteolytic enzymes, which have the ability to degrade the pulmonary extracellular matrix (ECM) by facilitating cell migration and activating growth factors [120]. The MMP-9/12 inhibitor AZ11557272 has been shown to be efficacious in a guinea pig cigarette smoke-induced emphysema model, but the compound was found to be toxic [57]. Although selective inhibitors of MMP-9 and -12 appear to be efficacious for the treatment of COPD in animal models, they have not been effective in clinical trials of COPD [59]. Neutrophil elastase inhibitors are also potential therapeutic drugs that can (i) degrade the ECM and proteins, (ii) destroy the lung parenchyma, and (iii) control the exuberant inflammatory response [121]. Several neutrophil elastase inhibitors have been tested in clinical development, but some of them have been withdrawn from clinical testing for various reasons [122].

### 4.3. Kinase Inhibitors

#### 4.3.1. p38 MAPK Inhibitors

The p38 mitogen-activated protein kinase (MAPK) pathway is activated by cellular stress, and it regulates the expression of a number of inflammatory proteins that are involved in COPD and asthma, e.g., CXCL8, IL-1β, TNF, and MMP-9. The p38 MAPK is activated in alveolar macrophages, lymphocytes, epithelial cells, and endothelial cells [123]. For example, the p38 MAPK inhibitor BIRB-796 showed enhanced anti-inflammatory effects on lipopolysaccharide-mediated cytokine production by alveolar macrophages, isolated from patients with COPD and smokers. Corticosteroids are ineffective in these patients [80]. Several p38 MAPK inhibitors are in clinical development for the treatment of COPD (Table 2). In patients with COPD, the oral p38 MAPK inhibitor losmapimod GW856553 was well tolerated and reduced plasma fibrinogen levels by 11% over a 12-week period, but there were no significant effects on lung function or sputum neutrophils [83]. Although p38 MAPK inhibitors have been tested in clinical trials of COPD and asthma, none of them have advanced into phase 3 studies due to side effects and toxicity, e.g., (i) undesired pharmacological activity, (ii) suppression of the innate immune response to viral and bacterial infections, (iii) damage of the central nervous system and liver, and (iv) poor or transient efficacy [124,125]. p38 MAPK include four isoforms that are α, β, γ, and δ subgroups [126]. An alternative strategy is to reduce the selectivity of the inhibitors, or to use selective inhibitors of the α-δ subgroups. Efforts have increasingly turned toward the development of inhaled formulations of p38 MAPK inhibitors. An inhaled p38 α MAPK antisense oligonucleotide (ASO) was shown to suppress allergic inflammation in mice, which confirms the feasibility of local p38 α MAPK inhibition [86]. Another possibility is to develop drugs that target upstream or downstream substrates in the MAPK pathway. For example, apoptosis signal-regulating kinase 1 (ASK1, also referred to as MAP3K5) is upstream of p38 MAPK and 0020JNK, and it may be activated by cigarette smoke and oxidative stress, hence it constitutes a potential target for inhibition [127].

#### 4.3.2. PI3K Inhibitors

PI3K (classes I, II, and III) generates lipid second messengers that regulate various cellular events [128]. Total PI3K activity is determined by measuring the level of the phosphorylated downstream target AKT. The activity is markedly increased in peripheral lungs and macrophages of patients with COPD [129]. Class I PI3K is the most widely studied heterodimer, which is composed of a regulatory subunit (p85) and a catalytic subunit (either p110, p110α, p110β, p110δ, or p110γ), among which the PI3K δ and γ subtypes play important roles in inflammation [130,131]. PI3K δ activation contributes to corticosteroid resistance, and this subtype participates in the differentiation, activation, and migration of T cells and NK cells [130,132], whereas PI3K γ is proinflammatory and is involved in neutrophil migration [133]. In a murine model of COPD, aerosolized PI3K γ/ δ inhibitor (TG100-115) suppressed lung inflammation without causing severe side effects [90,134]. Targeting PI3Kδ has been suggested to be a potential therapeutic strategy for COPD treatment. The efficacy of the PI3Kδ inhibitor nemiralisib (GSK2269557) has been investigated in four phase 2 clinical trials (Clinical Trial No.: NCT02294734, NCT02130635, NCT02522299, and NCT03345407). Blocking the PI3Kγ signal pathway is also a prospective strategy. Investigation of such targeted therapies provide unique and valuable insights for addressing inflammatory diseases, but they are still undergoing preclinical evaluation [91].

### 4.4. RNA Therapeutics

RNA therapeutics are promising for the treatment of airway inflammation and hyperresponsiveness, and nucleic acid-based therapy has been suggested for therapy against COPD and asthma [135,136,137,138,139]. Compared to conventional therapeutic approaches using small-molecule drugs, peptides, proteins, and monoclonal antibodies, RNA-based therapy provides additional advantages, including rapid bioinformatics-based design, high selectivity and potency, and the possibility of providing personalized therapy [140,141]. RNA therapeutics include three different classes [142,143]: (i) drugs that inhibit gene expression (e.g., small interfering RNA (siRNA), ASOs, and microRNA (miRNA), Piwi-interacting RNA (piRNAs), long noncoding RNAs (LncRNAs), and circular RNAs (circRNAs)); (ii) protein-encoding drugs (e.g., mRNA); (iii) protein-targeting drugs (e.g., RNA aptamers). The key pathological feature of obstructive airway diseases is the involvement of multiple inflammatory mediators that are often regulated by key genes [144]. Hence, targeting these key proinflammatory genes simultaneously may potentially improve the overall treatment outcome.

The main class of RNA therapeutics investigated for obstructive airways diseases is siRNA, and, more recently, also miRNA. miRNAs are considered both as potential biomarkers and as new therapeutic targets [145]. Several potential miRNA therapeutics have been identified and validated in animal models of COPD and asthma, e.g., miR-146a [146], -145 [147], -17 mimics [148], -320 and -150-5p [149], -186 [150], 181a-2-3p [151], -197 [152], -503 [153], -483-5p [154], -183-5p and -3177-3p [155], -218-5p [156], -3202, -206,-195, -145-5p, -181c, -27-3p [143]. siRNA has been shown to be effective in targeting NF-kB and genes involved in (i) mucus secretion, (ii) COPD exacerbation, (iii) airway remodeling, and (iv) remodeling of pulmonary vasculature [143]. In contrast, LncRNA can act as a competing endogenous RNA (ceRNA) that competitively adsorbs miRNA to reduce the binding of miRNAs to the target genes, hence leading to changes in the expression of the target genes of the miRNA [157,158]. The safety and efficacy of ALN-RSV01, which is a siRNA that targets the nucleoprotein of the respiratory syncytial virus (RSV), has been demonstrated in clinical trials (Clinical Trial No. NCT00496821 and NCT01065935) [92,93], and inhaled ALN-RSV01 represents a milestone in inhaled RNA therapy. The expression profiles of miRNA and mRNA in COPD patients were associated with chronic mucus hypersecretion [159]. MRT5005 is an inhaled mRNA candidate for the treatment of cystic fibrosis, and it delivers mRNA encoding functional transmembrane conductance regulator protein (Clinical Trial No. NCT03375047) [94]. These clinical studies provide significant proof-of-concept for local administration of therapeutic RNA in managing obstructive airway diseases.

However, nucleic acids are negatively charged hydrophilic macromolecules, and naked DNA/RNA (without any delivery vectors) can usually not bind to the cell surface and permeate the cell membrane by passive diffusion. Furthermore, the use of nucleic acids as drugs are associated with additional challenges, such as rapid degradation by nucleases, off-target gene silencing, and immune-stimulatory effects, that also need to be resolved. Chemical modification is one of the most effective ways to improve the pharmacokinetics, pharmacodynamics, and/or biodistribution of RNA. Chemical modification of (i) the nucleic acid backbone, (ii) the sugar moiety of ribose, and/or (iii) the nucleobase are widely adopted to improve the drug properties of oligonucleotides [160]. Among the 12 oligonucleotide-based therapeutics approved to date, eight are naked, relying solely on chemical modification to facilitate their delivery [160]. The extensive chemical modification of second-generation gapmer ASOs is sufficient for delivery to various tissues without the need for additional delivery agents [161]. The delivery potential of ASOs and siRNAs can be enhanced through their direct covalent conjugation to various moieties, e.g., lipids, peptides, aptamers, antibodies, and sugars [160]. For obstructive airway diseases, pulmonary administration of nucleic acids is considered one of the major portals. It is difficult for RNA to transcend biological barrier of lungs and successfully reach deep lungs. Hence, the design of dry powder formulations should be carefully optimized to obtain a product of the desired powder quality. In addition, the selection of an appropriate carrier system is critical for safe and effective delivery of RNA therapeutics via inhalation.

## 5. Dry Powder-Based Inhaled Medicines

Dry powder inhalers have become increasingly attractive for pulmonary delivery of medications acting locally and/or systemically. Their advantages are evident in the popularity of inhaled therapies for treating obstructive airway diseases over the last decade. For example, the oral bioavailability of fluticasone propionate is generally less than 1%, while inhaled forms of fluticasone provides 10 times higher bioavailability with minimum side effects [162]. However, formulation development for dry powder inhalers is challenging. Several techniques have been used to prepare inhalable dry powders, which differ in production cost, stability, and compatibility with the active pharmaceutical ingredient (API). Manufacturing methods can be classified into top-down approaches and bottom-up approaches [163,164]. Top-down approaches involve the size-reduction of large particles to the micro/nanometer, and only meet basic quality requirement, e.g., jet milling. Top-down approaches involve high energy input, are highly inefficient, and more complex performance requirements are difficult to achieve [163]. Bottom-up approaches, e.g., (i) spray drying, (ii) spray freeze drying, (iii) supercritical fluid technology, and (iv) nonwetting templates (PRINT) that involve the assembly of molecular components. Therefore, bottom-up approaches display higher potential for quality-oriented dry powder manufacturing and more complex structure design can be obtained [165].

### 5.1. Milling

In the pharmaceutical industry, milling has been used extensively to produce particles suitable for inhalation. Crystallization processes usually result in polydisperse particles with average diameters above 10 µm, providing a need for further jet-milling into a suitable size range. Several types of jet mills exist, e.g., fluid impact mills, opposed jet mills, spiral jet mills, oval chamber jet mills, and fluidized bed opposed jet mills, which have been comprehensively reviewed elsewhere [166]. The jet milling technology reduces particle size by interparticle collision and friction. However, jet milling is a high-energy input technology that comminutes the particles by insertion of a pressurized grinding gas, which creates a turbulent flow, promoting particle–particle and particle–wall collisions. In addition, the use of this technique can be time-consuming and inefficient for some materials, and it is not particularly suited for production of particles with customized shape, density, and/or surface properties [167]. The process also influences the hydrophobicity and aerosol performance of the resulting dry powders. The jet milling process involves mechanical processing, e.g., crushing and grinding, which has been shown to affect the material crystallinity and has the potential for strong interparticle cohesive forces [167]. Hence, the process is not suitable for fragile molecules and more complex engineered structures, e.g., porous/hollow particles and aggregates, or surface-modified, coated, or encapsulated materials.

### 5.2. Spray Drying

Another frequently applied technique is spray drying, which is used to transform liquid dispersions into dry particles by spraying the liquid into a hot drying medium. The feed sample can be a solution or suspension containing the API, with or without excipient(s), and the solvent used for preparing the feed sample can be water, an aqueous solution, an organic solvent, or a cosolvent. The process parameters applied during spray drying affects the efficiency of the drying process and the properties of the final product. The two main stress factors involved in this process are heat and high shear forces, which may disrupt the particle structure and result in degradation. The negative impact of these stress factors can be minimized by carefully (i) adjusting the composition, (ii) selecting appropriate excipients, and (iii) optimizing the process parameters. Spray drying results in more spherical particles and a higher respirable fraction than mechanical micronization of drugs for example by jet milling [168]. However, the use of spray drying is limited by the restricted outlet temperature range for thermolabile drugs, which are prone to degradation [169]. Spray drying can not only be used to manufacture inhalable particles of small-molecule API and proteins/peptides, but it can also be used to manufacture inhalable microparticles of drug-loaded nanoparticles (nanoembedded microparticles).

### 5.3. Spray Freeze Drying

Spray freeze drying is a relatively new technique, which combines the freeze-drying and spray-drying processing steps [170]. Depending on the nozzle position in the spray freeze-drying process, the droplets are sprayed beneath or above the surface of a cryogenic fluid. A combination of microfluidics and spray freeze drying has been used to produce dry powders for inhalation [171]. Budesonide fine particles were prepared using a microfluidic reactor coupled with ultrasonic spray freeze drying, hence avoiding additional homogenization or use of a stabilizer. The resulting dry powder displayed a fine particle fraction (FPF) range 47.6–54.9%, thus exhibiting promising aerosol performance. In contrast to spray drying, spray freeze drying is conducted at subambient temperature, and it has therefore been used to formulate a significant number of thermosensitive active drug compounds into dry powder inhalation products. Particulate products manufactured by spray freeze drying display lighter and more porous particles than those prepared using the spray drying technique [172]. However, spray freeze drying is still faced with the limitation of stress associated with freezing and drying, which may cause irreversible damage to biomacromolecules. This is evident as structural denaturation, aggregation, and loss of biological activity upon reconstitution. As for spray drying, loss of stability due to unfolding and aggregation remains a major challenge. Furthermore, spray freeze drying is time-consuming and has safety issues related to spraying into cryogenic fluids, and it is a relatively expensive process.

### 5.4. Supercritical Fluid Technology

Supercritical fluid is a compressed gas or a liquid above its critical pressure and temperature. It has several advantages as a solvent or nonsolvent in pharmaceutical production. So far, carbon dioxide, which is a low-cost, nontoxic pharmaceutical preparation, has been used as the most attractive supercritical fluid, both as a solvent or as an antisolvent. The solubility of the drug powder in the supercritical fluid will depend on (i) the density of the fluid, (ii) the chemical structure of the drug, and (iii) the contact time between the supercritical fluid and the drug. The morphology and size distribution of the resulting particles depend on the solute’s pre-expansion concentration in the supercritical fluid and the expansion conditions (e.g., temperature and pressure). The nozzle must be maintained at a suitable pre-expansion temperature to prevent premature precipitation of the drug [173]. Supercritical fluid technology can also be used to formulate inhalable RNA/protein microparticles. siRNA-chitosan nanoparticles and doxorubicin hydrochloride were coloaded in poly-l-lactide porous microparticles using supercritical fluid technology. The dry powder had an aerodynamic diameter (D_a_) between 1 and 5 µm and a FPF above 50%, and the microparticles induced in vitro gene silencing [174]. Nanoparticles coloaded with siRNA and glucagon-like peptide-1, embedded in porous microparticles, were prepared using the supercritical carbon dioxide technology. The dry powder had a D_a_ of 4.7 µm and a FPF over 60%. The expression of dipeptidyl peptidase-4 mRNA was efficiently inhibited by siRNA, and the hypoglycemic activity of coloaded GLP-1 was shown to be improved in diabetic mice [175].

### 5.5. Non-Wetting Templates (PRINT)

Another new technique, which has been used for particle engineering, is the PRINT technology, also referred to as the ‘micro-mold’ technology. A mixture of the drug and excipient(s) is pressed into micro-molds with the desired particle size and shape, and the micro-molds are subsequently removed. Unlike other particle engineering technologies, the dry powders prepared using the PRINT technology display precisely defined and reproducible particle geometries and shapes. The chemical structure and bioactivity of biopharmaceuticals have also been shown to be well preserved [176]. Additionally, the PRINT technology has been adapted for roll-to-roll manufacturing to support preclinical and clinical studies. The PRINT technology has been used to produce dry powder microparticles with uniform shape and size and are currently undergoing a phase 1 clinical testing [177]. Two new inhaled formulations of ribavirin (Ribavirin-PRIN-CFI and Ribavirin-PRINT-IP) were developed to achieve efficient delivery to the lung and minimize bystander exposure [177]. The PRINT technology also shows potential for controlling powder chemical composition and aerodynamic size. However, the PRINT technology falls short with respect to fabricating particle shapes to achieve suitable aerodynamic properties by changing the dynamic shape factor.

## 6. Critical Quality Attributes of Inhalable Dry Powders

**Figure 4:**
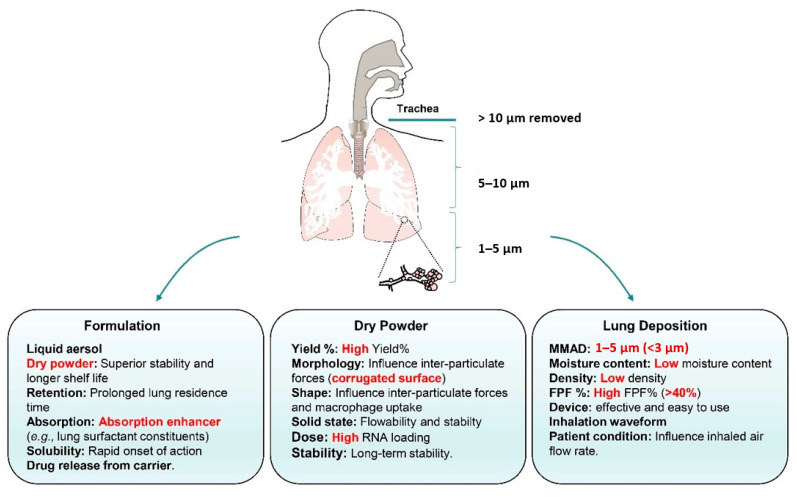
Critical quality attributes of inhalable dry powders.

Dry powders for pulmonary administration have to fulfill several criteria (Figure 4). The dry powder yield is evaluated by determining the mass of the recovered product, and it is calculated as the ratio between the mass of total recovered product and the mass initially fed into the system. The yield should be maximized from production and economic perspectives, and it is usually 20–70% for lab-scale production of spray-dried powders [178]. Powder loss can occur during expulsion of the drying gas or powder can be deposited on the wall of the manufacturing devices due to the cohesive nature of powders. For spray drying, the glass transition temperature (*Tg*) of the excipient displays a significant influence on powder yield [169]. For excipients with a low *Tg*, the powder yield is reduced, because the sticky powder is collected predominantly on the walls of the chamber or the cyclone. The process parameters used for spray drying also affect the powder yield. The yield is improved at higher feed flow rates for higher matrix solid concentrations, and lower feed flow rates for lower matrix solid concentrations [179]. Besides the influence on the powder yield, various spray drying parameters should be fully explored, because changing one parameter will affect multiple results, e.g., increasing the feed rate reduces the temperature within the drying chamber and increases the moisture content of the particles [180].

Particle aerosolization including aerosolization from the device, and subsequent deposition in the lungs, is another important factor for consideration for deep lung delivery. The aerosol performance of a dry powder is usually determined by applying impingers or impactors, e.g., the Anderson Cascade Impactor, the Multistage Liquid Impinger, or the Next Generation Impactor. The emitted dose (ED), the FPF, the fine particle dose (FPD), and the MMAD are the parameters used to evaluate the aerosol performance of dry powders. The MMAD can be reduced by (i) decreasing the geometric particle size, (ii) decreasing the particle density, or (iii) changing surface morphology. The physicochemical properties, e.g., moisture content, density, particle size, surface roughness, particle shape, solid-state properties, and interparticulate forces, are critical parameters that may be controlled or altered to achieve optimal performance with higher ED and FPF.

A low moisture content is a critical quality attribute of dry powders. The moisture content of dry powders may influence the powder stability and the aerosol performance through its effect on the particle-device interaction forces. A high residual moisture content influences the dispersion of the dry powder during aerosolization through capillary forces [181], while the influence of electrostatic forces could be considerable under dry conditions [182]. The moisture content is usually measured by thermogravimetric analysis (TGA) or Karl Fischer (KF) colorimetric titration. The moisture content is influenced by the properties of the excipient(s), e.g., powders prepared with mannitol have a relative low moisture content [183], dextran-based powders have a relative high moisture content [184], and amino acids as excipients can diminish the moisture content in the spray-dried powders [185]. The use of high inlet temperatures and feedstock concentrations, and nonaqueous solvents, lead to lower moisture contents, whereas high aspirator and feedstock rates have the opposite effect [186]. High outlet temperatures generally result in lower moisture contents in the recovered particles due to a more efficient drying process [187]. In addition, the use of organic solvent for spray drying generally results in inhalable powders with greatly reduced residual moisture content [188]. A secondary drying step can also be used to reduce the moisture content, if the dry powder has a high moisture content.

A low powder density is advantageous for pulmonary drug delivery, because it can improve the powder dispersibility and the drug delivery efficiency by reducing the MMAD of the dry powder, hence improving the aerosol performance. The density is calculated from the initial powder mass and the volume of the compressed powder. Large porous particles with a wrinkled morphology have a lower density. Shell formation can be forced by choosing a high initial saturation, a high Peclet number, or both. Internal and external void spaces can be created by choosing process conditions and formulations that lead to early separation of a soft surface layer [189]. Large porous particles with geometric diameters larger than 5 µm but with a low density of 0.1 g/cm^3^ were found to be highly inhalable and efficiently deposited in the lungs, which had respirable fraction particles of 50% and displayed approximately 10 times bioavailability than that of conventional inhaled particles [190]. Another study investigated the use of spray drying in the presence of a mixture of sugar (mannitol) and amino acids (L-leucine, glycine, and threonine) that eventually resulted in a powder with a low density of 0.14 g/cm^3^ [146]. The corrugated particles obtained in the study showed a high FPF of 51.3%. Another strategy to decrease the density of the dry powder is to design microparticles with a sponge-like morphology. The PulmoSphere^®^ technology, which is an emulsion-based spray-drying process was used to produce low-density particles with a high surface area and a sponge-like particle morphology [191]. For example, significant improvements in aerosolization performance of powders and a reduction in particle density were achieved by spray drying budesonide from ethanol/water or methanol/water solutions with ammonium carbonate as the pore-forming agent [192]. A carrier-based formulation can also be used to produce micronized drug particles that strongly adhere to the small porous PulmoSphere™ particles as respirable agglomerates [193].

Particle size is one of the most important quality attributes when aerosolizing dry powders, along with shape, density, and hygroscopicity. To reach the lower respiratory tract and for optimal pulmonary drug deposition, aerosols need to have an MMAD between 1 and 5 µm [27]. The particle size distribution is also a critical attribute of inhalation products, because it directly affects the site of deposition, and consequently the delivery efficiency. The particle size also influences the particle uptake in the lungs. Phagocytic uptake was shown to be reduced following inhalation of large (d_g_~8.5 µm) porous biodegradable polymer particles, compared to nonporous particles (d_g_ less than 5 µm), which displayed the same aerodynamic diameter (2–3 µm) [43]. 

The microparticle morphology influences the dry powder aerosolization behavior, e.g., (i) dry powder agglomeration, (ii) the size of the contact area between the microparticles, and (iii) the contact area between the particles and the inhaler device. The surface morphology also influences the interparticulate forces, e.g., van der Waals’ forces, which are responsible for particle–particle interactions during aerosolization of powders [194]. Compared to spherical particles, particles with a wrinkled surface morphology display a reduced surface area for interparticle interactions with increasing void size, which leads to weaker interparticle forces [195]. Such surface irregularities have been reported to improve the aerosolization properties of the inhalable particles by preventing particle agglomeration [196,197]. Different types of excipients or stabilizers, feed solvents, and adjusting the outlet drying temperature can affect the particle morphology, and changing the feed concentration and the atomization rate might result in particles with different degrees of surface corrugation. Hydrophobic amino acids, e.g., leucine, valine, and tryptophan, are commonly employed to improve powder dispersibility for pulmonary drug delivery [178,198]. For example, when two hydrophobic amino acids (leucine and tryptophan) and one hydrophilic amino acid (lysine) were used to stabilize simvastatin, the spray-dried simvastatin leucine (FPF% is 47.9%) and tryptophan (FPF% is 53.8%) exhibited better aerosol performance than the spray-dried simvastatin lysine (FPF% is 4.2%) [199]. When spray drying under constant operation conditions from different feed solvents, e.g., water–ethanol mixtures (50:50–0:100, *v/v*), the FPF (%) increased significantly with decreasing d_50%_, water and ethanol content (≥87.5% *v/v* ethanol) of the spray-dried samples [200]. Additionally, different degrees of surface corrugation could be achieved using different feed concentrations and atomization rates. Higher corrugation degrees were obtained using lower feed concentrations and air atomization rates (larger droplets) [201]. The dynamic shape factor is another property, which influences the aerodynamic diameter and the aerosol performance. A pollen shape of particles can enhance the aerosolization and deposition properties, compared to particles having a similar volume equivalent diameter [202]. The FPF (%) of an elongated dry powder was shown to be significantly higher than that of spherical dry powders [203]. Macrophage uptake is also influenced by particle shape. Physical resistance by the particles is required for phagocytosis to take place, and hence stiff particles are more easily phagocytosed than soft particles [204]. The aspect ratio of particles greatly affects the particle uptake by alveolar macrophages, and aspheric particles are poorly taken up [43]. Hence, increasing the elongation ratio (ER) of the microparticles, e.g., needle-like particles, can increase the powder yield delivered to the lungs, which might relate to the increased flowing time [205]. However, the underlying mechanisms of the ER effect need further investigation.

The solid-state properties also influence both the aerosol performance and the storage stability. The effects of crystallinity on aerosol performance are complex, because crystallinity changes also affect other properties, including microparticle shape, electrostatic charge, and surface energy. Inhalable particles containing drugs or excipients in amorphous states may display other problems, because they can absorb atmospheric moisture, which may cause powder aggregation and recrystallization, eventually affecting the aerosol performance [206,207]. Hence, it is essential to understand the solid-state characteristics of dry powders during manufacture and storage.

For dry powder-based RNA therapeutics, the dose has to be taken into consideration for effective drug delivery. For example, a high dose of siRNA is required owing to a short half-life and poor targeting ability. The maximum amount of dry powder that can be inhaled each time by an average human being is limited, e.g., 20–30 mg is considered as a high dose [208]. The main limitation of RNA-based dry powders is that the amount of RNA loading per carrier is limited owing to the macromolecular nature of RNA. For nanoembedded microparticles, the loading of RNA is relatively low, but the efficacy of RNA has been improved by the use of drug delivery systems [209]. It is desirable to increase the RNA loading, as this could potentially allow for increasing the dose of RNA without changing the total powder mass [210]. It is unclear whether RNA-based carriers influence the aerosol performance of dry powders. The large amount of excipients required for dry powder formulations makes it difficult to prepare high-dose formulations. The highest loading of siRNA per dry powder mass prepared by spray drying was 2–6% (*w/w*) [211], and 6% (*w/w*) loading of siRNA per dry powder mass could be achieved by spray freeze drying [212].

Dry powder stability is critical to ensure the delivery of a reproducible dose to the airways. The physical stability of dry powder is closely related to the physicochemical properties mentioned above, e.g., the moisture content and the solid-state properties. Various particle engineering methods and excipients can be used to optimize the physical stability of dry powders [206], including moisture content, density, particle size, surface roughness, particle shape, solid-state properties, interparticulate forces, and aerosol performance. The chemical integrity of RNA therapeutics after spray drying, spray freeze drying, or supercritical fluid technology can be well protected, however, there are few reports on the long-term stability of RNA therapeutics. Spray-dried powders containing siRNA/peptides and the excipient mannitol have been shown to be stable in the crystalline form for up to 5 months of storage at 4 °C [213]. However, the stability of long single-stranded RNA is poor and requires adequate storage conditions.

## 7. Delivery Systems for Inhaled RNA Therapeutics

As described in Section 4.4, the biggest hurdle in the clinical translation of RNA remains identifying a safe and effective delivery system. Many delivery systems have been developed for pulmonary delivery of RNA. The major function of these delivery systems is to facilitate the uptake of RNA by target cells and to protect RNA from premature degradation. RNA therapeutics can be delivered as a naked RNA powder formulation or in the form of nanoembedded microparticles (Table 3). The nanoembedded microparticle-based delivery systems for pulmonary delivery can be classified as lipid-, polymer-, lipid-polymer-, and peptide-based delivery systems. More recently, exosomes, spherical nucleic acids and DNA nanostructures have been successfully used in delivering RNA to specific organs or tissues, but investigations of these delivery systems for pulmonary delivery are limited [160].

### 7.1. Microparticles

With the promising effect of naked RNA in eliciting gene silencing in vivo without the use of transfection agent in the airways, dry inhalable powders were developed to deliver RNA [141,142]. The major challenge for developing inhalable powder formulations of RNA therapeutics is to preserve the chemical integrity of the nucleic acid during the drying process, and the powders must display aerosol properties suitable for inhalation [214]. In addition, microparticles are used as vehicles for carrying nanoparticles loaded with RNA therapeutics into the lungs. Excipients constitute more than 99% of the dry powder mass, and they must be carefully selected for specific physicochemical properties of the resulting dry powders. For example, mannitol was used as a bulking agent and L-leucine as a dispersion enhancer for preparing inhalable naked siRNA-containing dry powders by spray drying [217], and the integrity of the siRNA was shown to be preserved during the drying process. In addition, the siRNA microparticles appeared to be highly porous, the structural integrity was well maintained, and the gene-silencing effect of the siRNA was preserved [217]. Biologically active siRNA dry powder for inhalation has also been prepared using supercritical fluid drying applying chitosan and mannitol as excipients with an siRNA loading of 2% (*w/w*) [216]. However, the powder displayed a long needle-like morphology and poor dispensability, which is not suitable for pulmonary delivery. The dry powders were also optimized using PEI (with high positive charge density) as a carrier and L-leucine as excipient by using the supercritical fluid technology. These inhalable siRNA dry powders had a high aerosol performance and pulmonary gene silencing activity [225]. There is no clear correlation between the chemical integrity and biological activity of spray-dried RNA powders. However, the chemical integrity of siRNA during the spray drying process is influenced by thermal and shear stresses, and up to 80% of intact siRNA was shown to be preserved under relatively harsh spray drying conditions [214]. However, the spray-dried siRNA processed under the extreme conditions displayed cellular transfection efficiency comparable to that of untreated siRNA. As the size and charge of DNA/RNA hinder membrane passage, the delivery of RNA to the target site in the cell cytosol is difficult. For effective delivery of RNA, the application of nanoparticle-based delivery systems, such as encapsulation into nanoparticles and electrostatic complex formation with liposomes or polymers before the preparation of dry powder, is promising. Drug delivery systems can not only avoid macrophage clearance because of the size of nanoparticles, but also enhance the cellular uptake of RNA via endocytosis, escape their degradation by nucleases in the body, and protect their integrity from stresses during powderization [233].

### 7.2. Nanoembedded Microparticles

Nanoembedded microparticles combine the merits of nanoparticles and the pulmonary delivery convenience of microparticles. The nanoparticles loaded with RNA therapeutics are suspended in an aqueous excipient solution and subsequently aerosolized into small droplets and dried, resulting in microparticles in which the nanoparticles are embedded. Under physiological conditions, nanoembedded microparticles redisperse into nanoparticles, allowing for controlled release of the encapsulated drug [234]. A proper nanoparticle encapsulation and protection is needed for minimizing RNA degradation and facilitates cellular uptake [9]. Such nanoembedded microparticles can maintain the structure of nanoparticles after the drying process and translate them into inhalable microparticle powder. Nanoembedded microparticles are attractive for the delivery of nanoparticles loaded with RNA cargoes to the respiratory tract, and several research groups have studied them. Ongoing research mainly focusses on designing suitable nanocarriers for delivery of RNA therapeutics to improve transfection in the airways (Table 3), whereas, the development of microparticles is still in its infancy.

#### 7.2.1. Lipid-Based Delivery Systems

Lipid-based delivery systems can be used to entrap hydrophobic and hydrophilic drugs to improve the pharmacological action and the pharmacokinetic profile. Lipid-based delivery systems can be classified into five categories: cationic lipoplexes and liposomes; PEGylated lipids; neutral lipids; lipids particles; and lipid-like molecules [235]. Charged cationic lipids form complexes with negatively charged DNA/RNA through attractive electrostatic interactions, i.e., the so-called lipoplexes. Lipoplexes bind to the cell plasma membrane and are endocytosed, which induce significant target gene knockdown. The major problems with lipoplexes is their toxicity and their ability to induce inflammation. Since the adverse effects are mainly due to the positive charge of lipids, most strategies to overcome the drawbacks of cationic lipid concentrate on shielding the positive charge with polyethylene glycol (PEG) [236], or by switching to a neutral lipid cholesterol [231]. Lipopolyamine/siRNA nanocomplexes modified with methoxy PEG (mPEG) showed significant target gene knockdown in lungs of healthy mice. The observed clearance rate from the lung tissue was slower than in other tissues, resulting in prolonged siRNA accumulation on the timescale of siRNA-mediated transcript depletion after intravenous injection [218]. Such applications will require further development of these delivery systems and a greater understanding of the underlying mechanisms dictating biodistribution and retention of nanocomplexes. Freeze drying has been used to conserve the gene-silencing efficiency of formulated RNA and the supramolecular structure of the lipid particulate system. The results showed that the drying process was successful, but the sample flowability was not suitable for the inhalation usage, and the powders were further compressed into tablets [220]. Different concentrations of sucrose, trehalose or mannitol have been used to improve the stability of mRNA-loaded lipid-like nanoparticles. Nanoparticles could retain their mRNA delivery efficiency for at least three months through in vitro and in vivo mRNA delivery studies. The experiment shows the possibility of using the spray freeze drying method to prepare dry powder for inhalation [237]. However, there are few studies on the use of lipoplexes as inhaled formulations.

#### 7.2.2. Polymer-Based Delivery Systems

Nanoparticles have potential applications in diagnosing diseases and drug delivery due to their controlled drug release, target specificity, and better therapeutic index [238]. The polymer-based delivery vectors change their physicochemical qualities to effectively deliver drugs. Polymers usually do not induce a strong immune response, taking one step closer to clinical application. The majority of the polymers applied for RNA delivery including poly(d,l-lactide-co-glycolide) (PLGA), poly(glycerol adipate-co-ω-pentadecalactone) (PGA-co-PDL), PEI, chitosan, and their derivatives.

Synthetic polymers may be employed to enhance delivery of RNA therapeutics to various tissues. Polymers can form polyplexes through spontaneous electrostatic interaction. The size of polyplexes is usually in the range of 50–200 nm, and it is affected by the molecular weight of polymers, the charge ratio, and the pH value and ionic strength of the medium [239]. A pulmonary siRNA delivery system based on transferrin-polyethyleneimine (Tf-PEI) was prepared to avoid potential systemic side effects and increase the selectivity for activated T cells for asthma therapy. The prepared Tf-PEI polyplexes were in a range of 72–197 nm and successfully induced gene knockdown in a murine model of allergic asthma. Repeated administration of Tf-PEI polyplexes was well tolerated in healthy animals and no toxicity was observed [221]. Inhalable PEI-based dry powder shows the possibility for inhalation therapy as we mentioned in Section 5.1 [225]. PEI has also been used as carrier for miRNA along with chitosan. The results show that the uptake of miRNA is highly polymer-dependent, but the experiment did not show a direct correlation between the levels of miRNA and the downstream gene knockdown [223]. Polyplexes can effectively deliver RNA therapeutics, however, the clinical application of PEI or its conjugates is still limited due to the concerns of possible toxicity.

A series of dendrimer vectors have been used for RNA delivery [126,158]. The most commonly used polymeric materials for preparing dendrimers include poly(amidoamine) (PAMAM), poly(l-lysine) (PLL), polyamides, polyesters (PGLSA-OH), polypropylenimine (PPI), poly(2,2-bis(hydroxyl methyl) propionic acid), and polyethers [240]. Cationic dendrimers, e.g., PAMAM, was used for siRNA delivery to achieve high transfection efficiency. A 3D-printed micromixer was used for the preparation of siRNA-PAMAM dendrimer [136]; subsequently, dendrimer was processed into microparticle-based dry powders for inhalation using spray drying with trehalose and inulin as excipients. The result showed that the integrity and gene silencing efficiency of siRNA was preserved in the reconstituted nanocomplexes. Inhalable RNA-loaded nano-embedded microparticles can be engineered using microfluidics and spray drying. PAMAM was modified with triphenylphosphonium (TPP) (G4NH2-TPP, generation 4) to prepare dendrimers with various TPP densities and N/P ratios. The gene knockdown ability of the TPP-dendriplexes was affected by the TPP density. The transfection ability could also be potentially increased if an increase of TPP density on dendrimer were to occur, so there is a balance between potential toxic effect and TPP modification. Dendriplex with 12 TPP and at 30 N/P ratios emerged as the most promising formulation. The dry powder of this formulation with mannitol as excipient had aerosol characteristics that were conducive for deep lung deposition and had no impact on the transfection efficiency of siRNA [224]. Dendrimer are usually criticized by relatively low transfection efficiency and non-negligible toxicity. In addition, complexes based on electrostatic interactions tend to disassemble or aggregate over time because of the possible interaction with the airway surface liquid, which are negatively charged [239]. Therefore, dendrimer generation, architecture, surface functionality, and the core play important roles in RNA delivery.

Solid polymeric nanoparticles may offer improved colloidal stability and controlled release of their payloads. The parameters of importance for spray drying of siRNA-loaded PLGA nanoparticles into nanocomposite microparticles for inhalation have been evaluated [226]. The optimal formulation produced with mannitol displayed a low water content (0.78% *w/w*) and an aerodynamic particle diameter 4.99 ± 0.15 µm was considered suitable for inhalation. The gene silencing activity of the siRNA formulation was later improved by employing cationic lipid-modified PLGA nanoparticles in the spray-dried formulations. Solid nano-in-nanoparticles (double nano carriers (DNCs)) consisting of siRNA-loaded serum albumin nanoparticles that were used as a core, and PLGA that was used as a wall material, was prepared by the innovative technology of nano spray drying. DNCs (with a median size of 580–770 nm) were produced by spraying at low temperatures (less than 60 °C) to prevent damage to heat-sensitive siRNA and the silencing activity of double encapsulated siRNA extracted from DNCs was fully preserved [227]. Spray dried Bac-TMC3/TPP/siRNA nanoparticles into mannitol microparticles possessed good aerodynamic properties (FPF% is 45.4%) for deep lung deposition and pulmonary delivery of siRNA avoided the serum-induced degradation [228]. Spray drying with the proper selection of excipients and mild processing conditions is likely a promising strategy to preserve the physicochemical properties and biological activity of RNA.

#### 7.2.3. Lipid-Polymer Hybrid Delivery Systems

Lipid-polymer hybrid nanoparticles (LPNs) are core-shell nanoparticle structures comprising polymer cores, e.g., PGA-co-PDL or PLGA, and lipid/lipid-PEG shells, which exhibit complementary characteristics of both lipid and polymeric nanoparticles, particularly in terms of their physical stability, transfection efficiency, and biocompatibility. LPNs have the potential to enhance physical stability and biocompatibility of encapsulated RNA. miR-146a loaded PGA-co-PDL-DOTAP NPs were prepared to resist COPD pathogenesis by targeting interleukin-1 receptor-associated kinase 1 (IRAK1) expression and reducing IL-8 promoter reporter GFP via IL-1β signaling pathway [229]. miR-146a retained biological activity in vitro with 40% reduced IRAK1 expression and reduced IL-8 promoter reporter GFP. Similar experiment was performed using PLGA and cationic lipids (DOTAP) as carriers for siRNA and spray dried into microparticles with mannitol [241]. The processing conditions were found to preserve the integrity of the siRNA. To further improve the toxicity issues of DOTAP, a new synthetic lipid-like material termed lipidoids was employed to enhance the efficacy of siRNA [242]. A systematic quality-by-design approach was used to define the optimal formulation parameters [138]. The optimized lipidoid-modified LPNs revealed more than 50-fold higher than DOTAP LPNs in vitro gene silencing at well-tolerated doses than the commercial lipofectamine. Moreover, the LPNs microparticles had an MMAD suitable for lung deposition after spray drying using mannitol as the stabilizing excipient [209]. Dispersed nanoembedded LPNs had preserved physicochemical characteristics, as well as in vitro siRNA release profile and gene silencing. Apart from dry powder, nebulization is able to delivery miR-17 LPNs for inhalation with high FPF% (89.9%) [148]. These results show highly promising prospects for efficient and safe intracellular delivery of RNA and suggest that it is worth putting more efforts in promoting this vector for the clinical application of therapeutic RNA. In order to achieve better in vivo performance of dry powder aerosols upon inhalation in preclinical animal models of inflammation, the flowability of the dry powder aerosols should also be taken into consideration.

#### 7.2.4. Peptide-Based Delivery Systems

Peptide-based delivery systems are another promising platform for pulmonary delivery of RNA since the discovery of cell-penetrating HIV-1 TAT peptide, which assist in the uptake of the virus in the cell [243]. Pulmonary surfactants could facilitate the delivery of polymer-based delivery systems [244]. The transfection efficiency of siRNA in alveolar macrophages was improved in mice following pulmonary administration by coating pulmonary surfactant on siRNA-loaded nano-gels [245]. A mimic of surfactant protein B, KL4 was used to load siRNA by forming nanosized peptide-based complexes. KL4/siRNA complexes remained stable and mediated efficient siRNA transfection in vitro in human lung epithelial cells, A549 cells, and BEAS-2B cells. Additionally, KL4 peptide is not toxic or immunogenic at tested concentrations [230]. A range of cell-penetrating peptides (CPPs) and derivatives have been identified to load macromolecules and facilitate the membrane translocation of RNA. CPPs also could be used to modify lipid- or polymer-based RNA delivery systems, e.g., the conjugation of octa-arginine and TAT to neutral lipids increased cellular uptake of siRNA and obtained 30–45% gene knockdown [231]. However, since both TAT and peptides alone induced changes in gene expression, these results need to continue to elucidate the mechanisms of CPP bioactivity. CPP-siRNA complexes condensed by calcium promisingly offer high (up to 93%) target gene silencing effects in the human epithelial lung cell line (A549-luc-C8) with little to no evidence of cytotoxicity [246]. PEG12KL4 peptide has been used as an mRNA delivery vector at 10:1 ratio (*w/w*), which were formulated into dry powders with high aerosol performance (FPF% of spray drying was 41% and of spray freeze drying was 68% at 0.5% mRNA loading) and had preserved biological activity. This is the first study that demonstrated in vivo transfection efficiency of inhalable dry powder mRNA formulations [232]. The peptides are either covalently connected to RNA through disulfide bond development or electrostatically in a noncovalent way [247]. However, very few studies on the design of inhalable peptide-based RNA formulations have been reported.

## 8. Conclusions and Perspectives

Obstructive airway diseases such as COPD and asthma are associated with chronic bronchitis and emphysema and are characterized by persistent airway inflammation, long-term breathing problems, and poor airflow, which ultimately lead to a gradual progression of irreversible airway obstruction. The standard approaches for the treatment of COPD and asthma, e.g., inhaled corticosteroids and bronchodilators, have significant therapeutic effects. However, they are not sufficient to decrease mortality, and they cause side effects such as infection, exacerbation of airway inflammation, and gastrointestinal adverse effects. The pathogenesis of obstructive airway diseases is mainly related to (i) an oxidation–antioxidation imbalance, (ii) a protease/antiprotease imbalance, (iii) overexpression of inflammatory mediators and cytokines, and (iv) activation of inflammatory signaling pathways (p38 MAPK and PI3Ks). Novel inhalable medicines, which can inhibit these target molecules, e.g., antioxidants, mediator antagonists, kinase inhibitors, and RNA, have been shown to be effective in preclinical and/or clinical trials. However, the translation of some of these medicines is challenged by nonsignificant improvements in respiratory symptoms and/or side effects. The effects and mechanisms of novel inhaled medicines targeting obstructive airway diseases are under evaluation. Drug delivery by inhalation represents a noninvasive means of administration, but particles must overcome lung-geometry and physiological barriers. To improve delivery and clinical efficacy, inhalable dry powder formulations with appropriate physicochemical properties, e.g., high aerosol performance, low moisture content, low density, and suitable particle size and morphology, are required. The advantages of inhaled microparticles (dry powders) in the treatment of obstructive airway diseases are obvious, and research in this area may intensify in the near future. 

RNA therapeutics are promising for the treatment of obstructive airway diseases, because they can target multiple proinflammatory genes with high specificity and no or low immune responses. Hence, RNA therapeutics display promising prospects for the treatment of obstructive airway diseases, and they may become important therapeutic modalities for the treatment of COPD and asthma in the future.

The translation of drug delivery systems for clinical application of RNA therapeutics has recently gained momentum, but is still in its infancy. Major obstacles for clinical translation of inhalable RNA therapeutics are as follows: (i) the limited amount of RNA, which can be loaded per amount of carrier material. (ii) Basic and clinical research in mRNA vaccines for the prevention of COVID-19 is currently blooming. However, the storage stability of long single-stranded RNA is generally poor and requires adequate storage conditions. (iii) The preclinical aerosol performance of dry powders determined in vivo in healthy animals rarely reflects the aerosol performance in animal models of lung diseases. (iv) Animal models of lung diseases often poorly reflect the pathophysiology of human diseases. For example, a murine allergic airway inflammation model has been used for preclinical studies of asthma. However, mice and humans display different distribution of lung inflammation [142]. (v) Animal models for obstructive airway diseases are developed using various chemical stimuli, e.g., cigarette smoke, allergen, or irritant gas exposures. However, the use of a single stimulus does usually not mimic the complex exposure of human lung tissue (multiple stimuli and variable stimulation time points) and the disease chronicity observed in real life [248]. (vi) In addition to acute toxicity tests, the long-term safety of RNA therapeutics should be evaluated carefully before clinical translation. (vii) Additional development costs, compared to injectable liquid formulations and oral solid dosage forms, pose a major obstacle to the translation of RNA-based dry powders. Therefore, the potential of RNA-based inhalable medicines for obstructive airway diseases should be explored in in vitro and in vivo models for pharmacodynamics, distribution, safety, and efficacy.

## Figures and Tables

**Table 1:** Examples of approved medicines used in the management of COPD and asthma.

	Mode of Action	Representative Drugs	Adverse Effects	Ref.
Corticosteroids	Corticosteroids bind to GRs in the target cell cytosol. The corticosteroid–GR complex binds to specific sequences on the upstream regulatory elements of target genes after translocation into the nucleus. GRs also interact with protein transcription factors and coactivator molecules in the nucleus, which regulate gene expression at a transcriptional level.	Oral corticosteroids (e.g., prednisone and prednisolone).	Fluid retention, increased appetite, weight gain, osteoporosis, capillary fragility, hypertension, peptic ulceration, diabetes mellitus, cataract, and psychosis (long-term oral corticosteroid therapy). Fail to reduce disease progression or mortality in COPD patients. High doses of ICSs increase the risk of pneumonia in most patients with COPD.	[14]
		ICSs, e.g., beclomethasone dipropionate, budesonide, ciclesonide, flunisolide, fluticasone, and mometasone.		
β2 adrenoceptor agonists	Act via specific receptors (ADRβ2), which are localized mainly on airway smooth muscle cells. Binding to ADRβ2 by agonists causes activation of the Gs-adenylyl cyclase ecAMPePKA pathway, leading to bronchial smooth muscle relaxation.	SABA, e.g., salbutamol, and terbutaline.	Lack of selectivity for the β2 AR, resulting in “off-target” effects mediated by either α or β1 AR, or ill-defined β2 AR-mediated effects that appear to involve either β2 AR desensitization, or exacerbation of airway inflammation and its consequences.	[19,20]
		LABA, e.g., formoterol, and salmeterol.		
Muscarinic receptor antagonists	Inhibit acetylcholine stimulation of muscarinic receptors, block airway smooth muscle contraction, and vagally induce increased mucus secretion.	SAMA, e.g., ipratropium bromide, and oxitropium bromide.	Dry mouth, bitter taste, and urinary retention. Systemic adverse effects are uncommon.	[21]
		LAMA, e.g., aclidinium bromide, tiotropium bromide, glycopyrronium bromide, and umeclidinium bromide.		
Antileuko-trienes	Inhibit 5-lipoxygenase, prevent leukotriene synthesis, inhibit LTD4 binding to its receptor, and prevent its action.	Oral. Leukotriene-receptor antagonist zafirlukast. Leukotriene-synthesis inhibitor zileuton antileukotriene, montelukast.	Uncommon.	[22]
Antibiotics	Inhibit NF-κB and other transcription factors, resulting in reduction of chronic inflammation. The precise mechanism of action has not yet been determined.	Macrolides, e.g., erythromycin, azithromycin, and telithromycin.	Nausea and diarrhea are the most common gastrointestinal adverse effects. Macrolides prolong the corrected QT intervals on electrocardiograms, which increases the risk of torsades de pointes, potentially resulting in ventricular fibrillation and sudden death. Telithromycin rarely causes liver injury, with high morbidity and mortality rates.	[23,24]
Cromones	Delay activation of chloride channels in cell membranes. Inhibit cell activation. Inhibit both antigen- and exercise-induced asthma.	E.g., inhaled sodium cromoglicate, and nedocromil sodium.	Poorly absorbed, and serious adverse effects are rare.	[25]

Abbreviations: ADRβ2: Adrenoceptor β2; AR: adrenergic receptor; GR: glucocorticoid receptor; ICSs: inhaled corticosteroids; LABA: long-acting β-agonist; LAMA: long-acting muscarinic receptor antagonists; LTD4: Leukotriene D4; NF-κB: nuclear factor kappa light chain enhancer of activated B cells; SABA: short-acting β-agonists; SAMA: short-acting muscarinic receptor antagonists.

**Table 2:** Novel medicines in preclinical studies and clinical trials for the treatment of obstructive airway diseases.

Novel Inhaled Medicine	Drug	Mechanisms/Effects	Administration Route	ClinicalTrials.gov Identifier	Phase	Main Finding	Ref.
Antioxidants	NAC/glutamines	A reactive oxygen species scavenger and precursor of reducing glutathione	Oral	NCT01136239	Phase 4	Completed. High-dose NAC (600 mg bid) reduced COPD exacerbations and improved small airways function.	[51,52,53,54]
				NCT00184977	Phase 4	Completed. Reduced the degree of deterioration in GOLDII-III COPD patients; high-dose NAC was not significantly beneficial in low-risk patients (600 mg/day)	
				NCT00476736	Phase 4	Unknown. High-dose NAC (1200 mg daily) reduced gas trapping and improved exercise endurance in patients with emphysematous COPD.	
	Catalase/SOD/GPx	Reduce ROS				Anti-inflammatory effects on smoking-induced lung inflammation in animal models	[55]
	Sulforaphane	Increases the gene expression of Nrf2	Oral	NCT01335971	Phase 2	Completed. Sulforaphanetrial in COPD patients did not induce the expression of Nrf2 genes or affect the level of other antioxidants	[56]
Protease inhibitors (MMPs)	AZ11557272	MMP-9/12 inhibitor. Ameliorates emphysema				Ameliorate morphological emphysema, small airways remodeling, and the functional consequences of these lesions in a non-murine species	[57]
	Multiple MMPs (MMP-1, -2, -3, -8, -9, and -10); cytokines (IL-6 and IL-8)	Degrade the extracellular matrix and drive tissue remodeling		NCT01701869		Completed. MMP-3, -7, -8, -9, -10, and -12 concentrations closely associated with CT markers of small airways disease. Emphysema severity was also associated with MMP-3, -7 and -10. No strong relationships between MMPs and the bronchial wall thickness of larger airways	[58]
	AZD1236	MMP-9/12 inhibitor. Inhibits emphysema	Oral	NCT00758706	Phase 2	Completed. No clinical efficacy of AZD1236 was demonstrated	[59]
	FP-025	MMP-12 inhibitor. Allergen-induced airway responses, inflammation, and remodeling		NCT03858686	Phase 2	Recruiting	
Protease inhibitors (neutrophil elastase)	Sivelestat (ONO-5046)	Protect the lungs from tissue damage and control the exuberant inflammatory response		NCT00417326	Phase 2	Completed. ONO-5046 approved in Japan for the treatment of pneumonia and respiratory failure	
	AZD9668 combined with budesonide/formoterol	Reversible and selective inhibitor of neutrophil elastase	Oral	NCT01023516	Phase 2	Completed. AZD9668 did not significantly improve respiratory signs and symptoms	[60]
	Alvelestat (MPH966)	Bronchiolitis obliterans syndrome		NCT02669251	Phases 1 and 2	Recruiting	
Cytokines/chemokines receptor inhibitors	Canakinumab	Inhibition of IL-1β, for inflammation and cardiovascular risk	Subcutaneous	NCT02272946	Phase 2	Recruiting	
	Tocilizumab	Inhibition of IL-6, for rheumatoid arthritis and inflammation diseases	Subcutaneous	NCT03288584	Phase 2	Recruiting. Improved endothelial function led to a greater increase of effective myocardial function and reduced the inflammatory burden and oxidative stress	[61,62]
	Infliximab	Inhibition of TNF-α		NCT00056264	Phase 3	Completed. Patients with moderate to severe COPD did not benefit from treatment	[63]
	Etanercept	Inhibition of TNF-α	Subcutaneous	NCT00789997	Phases 2 and 3	Completed. Etanercept is not more efficacious than prednisone for the treatment of COPD deterioration	
	Mepolizumab	Inhibition of IL-5	Intravenous or subcutaneous	NCT01691521	Phase 3	Completed. Significantly reduced asthma exacerbations	[64]
	Reslizumab	Inhibition of IL-5	Intravenous	NCT01287039 and NCT01285323	Phase 3	Completed. The use of reslizumab in patients with asthma and elevated blood eosinophil counts	[65,66]
	Benralizumab	Inhibition of IL-5	Subcutaneous	NCT01238861	Phase 2	Completed. Benralizumab seems to reduce asthma exacerbations in adults with uncontrolled eosinophilic asthma and baseline blood eosinophils	[67]
	Dupilumab	Anti-IL-4 receptor α monoclonal antibody, inhibits IL-4 and IL-13 signaling	Subcutaneous	NCT01854047	Phase 2	Completed. Benefit patients with uncontrolled persistent asthma	[68,69,70,71]
	Dupilumab			NCT01312961	Phase 2	Completed. In patients with persistent, moderate-to-severe asthma, dupilumab therapy was associated with fewer asthma exacerbations with improved lung function and reduced Th2-associated inflammatory markers	
	Dupilumab			NCT02414854	Phase 3	Completed. Dupilumab reduced severe exacerbation rates, improved FEV1 and asthma control, and suppressed type 2 inflammatory biomarkers in patients with uncontrolled, moderate-to-severe asthma with or without evidence of allergic asthma	
Cytokines/chemokines receptor inhibitors	SB010 (DNAzyme)	Therapeutic targeting of GATA3, which is an important transcription factor of the Th2 pathway	Inhalation	NCT01743768	Phase 2	Completed. Treatment with SB010 significantly attenuated late and early asthmatic responses after allergen provocation in patients with allergic asthma. Biomarker analysis showed attenuation of Th2-regulated inflammatory responses	[72]
	Brodalumab (AMG 827)	Blocking IL-17 receptor signaling	Inhalation	NCT01199289	Phase 2	Completed. Ineffective in patients with severe asthma, although the subjects were not selected for neutrophilic inflammation	[73]
	AZD4818	Inhibition of CCR1	Inhalation	NCT00629239	Phase 2	Completed. Inhaled AZD4818 did not indicate a beneficial treatment effect	[74]
	AZD2423	Inhibition of CCR1		NCT01215279	phase 2	Completed	
	Navarixin (MK-7123)	Inhibition of CXCR2		NCT01006616 and NCT00441701	Phase 2	Terminated. Improvement in FEV1	[75]
	AZD5069	CXCR2 antagonist	Oral	NCT01233232	Phase 2	Completed. No safety issues and no increase in infection rates in dosage group compared with placebo	[76]
	Navarixin (SCH527123)	Binds with high affinities to human CXCR1 and CXCR2, which are the receptors for ligands including IL-8, GRO-α, and CXCL5	Oral	NCT01006161	Phase 2	Withdrawn	[77]
				NCT01068145	Phase 1	Terminated. SCH527123 caused significant attenuation of ozone-induced airway neutrophilia in healthy subjects	
Cytokines/chemokines receptor inhibitors	BIIL 284	Inhibition of LTB4 receptor		NCT02249247 and NCT02249338	Phase 2	Completed. No data published	
	Zileuton	Inhibition of 5-LO		NCT00493974	Phase 3	Terminated (lack of feasibility due to low recruitment). No significant improvement in the treatment of COPD patients with acute exacerbations	[78]
Inflammasome inhibitors	Canakinumab	A human anti-IL-1β monoclonal antibody	Intravenous infusion	NCT00581945	Phases 1 and 2	Completed. No statistical differences in FEV1 and FVC among canakinumab-treated and placebo-treated COPD patients	
	MEDI8968	Inhibits IL receptor 1 (IL-1α and IL-1β)	Intravenous infusion	NCT01448850	Phase 2	Completed. MEDI8968 did not produce statistically significant improvements in AECOPD rate, lung function, or quality of life	[79]
	MEDI2338	A human anti-IL-18 monoclonal antibody	Intravenous infusion	NCT01322594	Phase 1	Completed. No statistical differences were observed between treated and placebo COPD patients	
Kinase inhibitors (p38 MAPK inhibitors)	BIRB-796 and dexamethasone	Inhibit p38 MAPK				p38 MAPK activation in alveolar macrophages is corticosteroid-insensitive; combining a p38 MAPK inhibitor with a corticosteroid synergistically enhanced the anti-inflammatory effects on LPS-mediated cytokine production by alveolar macrophages from patients with COPD	[80]
	Dilmapimod (SB-681323)	Inhibits p38 MAPK	Oral	NCT00564746, NCT00380133, NCT00439881	Phase 1	Completed. No results published	[79]
				NCT00144859	Phase 2	Completed. Inhibited TNF-α production after a single oral dose	
	Losmapimod (GW856553)	Inhibits p38 MAPK	oral	NCT02993757	Phase 2	Completed	[81,82,83]
				NCT01541852	Phase 2	Completed. Discontinued: not effective in COPD	
				NCT00642148	Phase 2	Completed. No significant effects on lung function or sputum neutrophils	
	Acumapimod (BCT197)	Inhibits p38 MAPK	Oral	NCT0133209	Phase 2	Completed. Well tolerated. Repeated single-dose acumapimod showed a clinically relevant improvement in FEV1 over placebo on day 8	[84]
	PH-797804	Inhibits p38 MAPK	Oral	NCT00559910	Phase 2	Completed. Significantly improved lung function and dyspnoea in moderate-to-severe COPD but was discontinued	[85]
	p38α-ASO	Reduces p38α MAPK mRNA expression	Inhalation			The ASO significantly reduced OVA-induced increases in total cell counts, eosinophil counts, and IL-4, IL-5, and IL-13 levels in bronchoalveolar lavage fluid	[86]
	PF-03715455	Inhibits p38 MAPK	Inhalation	NCT02219048 and NCT02366637	Phase 2	Terminated	
	AZD7624	Inhibits p38 MAPK	Inhalation	NCT02238483 and NCT02753764	Phase 2	Discontinued. Not effective in the treatment of COPD and asthma	[87]
	RV568	Inhibits p38MAPK pathway	Inhalation	NCT01661244, NCT01867762 and NCT01475292	Phases 1 and 2	Completed. Significantly increased FEV1 and reduced sputum malondialdehyde and serum myeloperoxidase in COPD patients	
Kinase inhibitors (P13K inhibitors)	Nemiralisib (GSK2269557)	Inhibits PI3Kδ	Inhalation	NCT02294734	Phase 2	Completed. Effective as placebo on FEV1	[88,89]
				NCT02130635	Phase 2	Completed. Acceptable safety profile for progression to larger study	
				NCT02522299	Phase 2	Completed. Did not significantly improve FEV1 and the use of rescue medication in patients with acute exacerbation	
				NCT03345407	Phase 2	Terminated. Unfavorable benefit–risk profile	
				NCT03189589	Phase 1	Completed. Progression to phase 2 study supported	
	TG100-115	Selectively blocks PI3Kγ and PI3Kδ	Inhalation			Inhibited pulmonary neutrophils induced by intranasal LPS and smoke in mice with COPD	[90]
	RV1729	Inhibits PI3Kδ		NCT02140346	Phase 1	Completed. Limited efficacy data have been collected	
	AS605240	Inhibits PI3Kγ	Oral	-	-	Prevented pulmonary fibrosis by suppressing inflammatory cell recruitment and production of inflammatory cytokines in bleomycin-induced pulmonary fibrosis	[91]
RNA therapeutics	ALN-RSV01	Regulating protein expression that is mediated by siRNA	Nasal spray	NCT00496821 NCT01065935	Phase 2	Completed. ALN-RSV01 has significant antiviral activity against human RSV infection	[92,93]
	MRT5005	mRNA encoding fully functional CFTR protein	Nebulization	NCT03375047	Phases 1 and 2	Recruiting. A marked improvement of lung function in patients after single dose at the mid-dose (8–16 mg) level	[94]
	Eluforsen	Single-stranded RNA ASO targeting CFTR	Intranasal in phase 1; Inhalation in phase 2	NCT02564354 and NCT02532764	Phases 1 and 2	Completed. Safe, well tolerated, and improved respiratory symptoms	

Abbreviations: ASO: antisense oligonucleotide; CCR: chemokine receptor; CFTR: transmembrane conductance regulator; CXCR: CXC-chemokine receptor; FEV1: forced expiratory volume in 1 s; FVC: forced vital capacity (the total exhaled breath); GATA3: GATA binding protein 3; GPx: glutathione peroxidase; GRO-α: growth regulated protein alpha; IL: interleukin; LTB4: leukotriene B4; 5-LO: arachidonate 5-lipoxygenase; LPS: lipopolysaccharide; MMP: matrix metalloproteinase; NAC: N-acetylcysteine; Nrf2: nuclear factor erythroid 2 (NFE2)-related factor 2; p38 MAPKs: mammalian p38 mitogen-activated protein kinases; OVA: ovalbumin; PI3K: phosphoinositide 3-kinase; ROS: reactive oxygen species; RSV: respiratory syncytial virus; SOD: superoxide dismutase; Th2: T helper type 2 cell; TNF-α: tumor necrosis factor α.

**Table 3:** Examples of dry powder-based formulations loaded with RNA for pulmonary delivery.

Type	Carrier	Drug	Key Excipients	Method of Preparation	z-Average (A) and MMAD (B)	Main Findings	Ref.
Microparticles	-	siRNA	Mannitol and HSA	Spray drying	B: 1.3–1.4 µm	Bioactivity of siRNA was preserved in RAW264.7 cells	[211]
	-	eGFP siRNA	Mannitol	Spray drying	-	HPLC was used to evaluate chemical stability of siRNA; the thermal and shear stress of the spray drying could influence the chemical integrity of siRNA	[214]
	-	MCP-1 siRNA	Mannitol	Spray freeze drying	B: 4.0–4.5 µm	Biologically active in RAW264.7 (mouse macrophage-like cells)	[215]
		siRNA	Chitosan and mannitol	Supercritical carbon dioxide technique	B: 10–20 µm	Biologically active in mice bearing colon 26/Luc cells	[216]
	-	siRNA targeting IL-10	L-leucine and mannitol	Spray drying	B: 1.35–1.99 µm	Examined the integrity of siRNA by gel retardation assay	[217]
Nanoembedded microparticles	Liposome	siRNA targeting GFP, CD31, CD45, and Tie-2	Lipopolyamine (Staramine)	Film-rehydration method	-	Slower clearance rate from the lung tissue and gene knockdown in the lungs of normal mice.	[218]
		siRNA specific to luciferase	1. DMAPAP and DOPE 2. Trehalose or trehalose with mannitol	1. Film-rehydration method [219] 2. Spray freeze drying	-	After compression into tablets, siRNAs retained more than 60% of their gene-silencing efficacy.	[220]
	Polyplexes	siRNA	Tf-PEI (molar ratio of Tf to PEI was 1.5:1)	Incubated after mixing Tf-PEI with siRNA	A: 72–197 nm	Successful gene silencing in a murine model of allergic asthma. Well tolerated in healthy animals and no toxicity	[221]
		VDBP- siRNA	DEXA-PEI	Incubated after mixing VDBP- siRNA with DEXA-PEI. Modified from [222]	-	Reduced goblet cell hyperplasia, ovalbumin sensitization, challenge-induced enhancement of airway inflammation, expressions of interleukin-4 (IL-4), IL-13, and eosinophil mobilizing chemokine (CCL11)	[137]
		MiR-126	PEI or chitosan	Mixed and left on ice for 30 min	A: From 100 to 1000 nm, depending on the N/P ratio and diluent solvents	miRNA uptake is highly polymer-dependent; no direct correlation between the levels of miRNA and the downstream gene knockdown	[223]
	Dendrimer	TNF-α siRNA	1. PAMAM 2. Trehalose and inulin	1. Bulk mixing and microfluidics 2. Spray drying	A: 87–103 nm B: ~5 µm	The integrity and gene silencing efficiency of siRNA was preserved	[136]
		eGFP siRNA	1. PAMAM 2. Mannitol	1. Vortex and incubate 2. Spray drying	A: 120–400 nm B: 3.8 ± 0.2 μm	Dendriplexes with the dendrimers containing the highest surface density of TPP and at N/P 30 showed the best gene knockdown efficiency	[224]
	Polymeric nanoparticles	siRNA	1. PEI 2. L-leucine	1. Incubated after mixing siRNA with PEI 2. Spray freeze drying	A: ~190 nm B: ~10 µm	In vivo pulmonary gene silencing	[225]
		siRNA	1. PLGA (molar ratio of 75:25, Mw: 20 kDa); 2. Trehalose, lactose, and mannitol.	1. Modified-SESD; 2. Spray drying	B: 4.99 ± 0.15 µm	Biologically active of spray-dried siRNA using H1299 cells	[226]
		siRNA	1. PLGA (molar ratio of 50:50, Mw: 100 kDa), DOTAP and HSA	1. Desolvation 2. Spray drying	A: 100 nm B: 580–770 nm by SEM	Biologically active of spray-dried siRNA using A549 cells	[227]
		Survivin siRNA	1. Bac-TMC, TPP 2. Mannitol	1. Formed spontaneously by electrostatic interaction 2. Spray drying	A: 232 nm B: 3.64 ± 0.06 µm	Biologically active of spray-dried siRNA using A549 cells	[228]
	LPNs	eGFP siRNA	1. PLGA, DOTAP, PVA 2. Mannitol	1. DESE 2. Spray drying	A: 261.1 nm B: 3.69 ± 0.18 µm	Preserve the integrity of the siRNA and the gene silencing activity of the siRNA-loaded PLGA nanoparticles.	[90]
		TNF-α siRNA	1. Penta-substituted lipidoid, PLGA, PVA 2. Mannitol	1. DESE 2. Spray drying	A: 197.1 ± 3.0 nm B: 3.3 ± 0.2 µm	The chemical stability of the siRNA was preserved upon spray drying with high loading. Data collected for mRNA expression after transfection in RAW 264.7 cells showed efficient gene silencing after spray drying	[138,209]
		miR-146a	PGA-co-PDL and DOTAP	Oil-in-water (o/w) single emulsion method	A: 244.8 ± 4.4 nm	miR-146a retained biological activity in vitro with 40% reduced IRAK1 expression and reduced IL-8 promoter reporter GFP	[229]
		miR-17	1. PLGA and DOTAP 2. Trehalose	1. DESE 2. Freeze drying	A: 208.0 ± 16.7 nm B: 4.20 ± 0.05 µm (nebulization)	Downregulated LPS-induced IL-8 secretion by >40% in bronchial epithelial cells	[148]
Nanoembedded Microparticles	Surfactant protein-based nanoparticles	siRNA	SP-B mimic, synthetic KL4 peptide	Incubated after mixing siRNA with KL14	A: 280–460 nm	It mediated siRNA transfection effectively in vitro in human lung epithelial cells, A549 cells, and BEAS-2B cells	[230]
	CPP based nanoparticles	siRNA	Cholesterol, TAT (48–60)	-	-	siRNA-mediated mRNA knockdown of p38 MAPK in mouse lung	[231]
		mRNA	1. PEG_12_KL4 2. Mannitol	1. Incubated after mixing siRNA with PEG_12_KL4 2. Spray drying or spray freeze drying	A: 467.9 ± 24.9 nm B: less than 5 µm	Effective transfection in the lung when administered intratracheally either as liquid or powder, with low risk of inflammatory response and toxicity	[232]

Abbreviations: Bac-TMC: baclofen functionalized trimethyl chitosan; DMAPAP: 2-{3-[bis-(3-amino-propyl)-amino]-propylamino}-N-ditetradecylcarbamoyl methyl-acetamide; DOPE: dioleoylphosphatidylethanolamine; DOTAP: cationic lipids; FPF: fine particle fraction; DESE: double emulsion solvent evaporation method; GFP: green fluorescent protein; HSA: human serum albumin; IRAK1: interleukin-1 receptor-associated kinase 1; LPNs: lipid–polymer hybrid nanoparticles; Modified-SESD: modified spontaneous emulsification solvent diffusion; PAMAM: poly(amidoamine); PEG: polyethylene glycol; PEI: polyethyleneimine; PLGA: poly(d,l-lactide-co-glycolide); PVA: polyvinyl acetate; SP-B: surfactant protein B; Tf-PEI: transferrin-polyethylenimine.

## Data Availability

Not applicable.
